# Nanocarrier Design for Dual-Targeted Therapy of In-Stent Restenosis

**DOI:** 10.3390/pharmaceutics16020188

**Published:** 2024-01-29

**Authors:** Ivan S. Alferiev, Kehan Zhang, Zoë Folchman-Wagner, Richard F. Adamo, David T. Guerrero, Ilia Fishbein, Danielle Soberman, Robert J. Levy, Michael Chorny

**Affiliations:** Division of Cardiology, The Children’s Hospital of Philadelphia, and Department of Pediatrics, Perelman School of Medicine, University of Pennsylvania, Philadelphia, PA 19104, USA

**Keywords:** magnetic guidance, restenosis, magnetic nanoparticles, affinity targeting, fibrin, paclitaxel, stent

## Abstract

The injury-triggered reocclusion (restenosis) of arteries treated with angioplasty to relieve atherosclerotic obstruction remains a challenge due to limitations of existing therapies. A combination of magnetic guidance and affinity-mediated arterial binding can pave the way to a new approach for treating restenosis by enabling efficient site-specific localization of therapeutic agents formulated in magnetizable nanoparticles (MNPs) and by maintaining their presence at the site of arterial injury throughout the vulnerability period of the disease. In these studies, we investigated a dual-targeted antirestenotic strategy using drug-loaded biodegradable MNPs, surface-modified with a fibrin-avid peptide to provide affinity for the injured arterial wall. The MNPs were characterized with regard to their magnetic properties, efficiency of surface functionalization, disassembly kinetics, and interaction with fibrin-coated substrates. The antiproliferative effects of MNPs formulated with paclitaxel were studied in vitro using a fetal cell line (A10) exhibiting the defining characteristics of neointimal smooth muscle cells. Animal studies examined the efficiency of combined (physical/affinity) MNP targeting to stented arteries in Sprague Dawley rats using fluorimetric analysis and fluorescent in vivo imaging. The antirestenotic effect of the dual-targeted therapy was determined in a rat model of in-stent restenosis 28 days post-treatment. The results showed that MNPs can be efficiently functionalized to exhibit a strong binding affinity using a simple two-step chemical process, without adversely affecting their size distribution, magnetic properties, or antiproliferative potency. Dual-targeted delivery strongly enhanced the localization and retention of MNPs in stented carotid arteries up to 7 days post-treatment, while minimizing redistribution of the carrier particles to peripheral tissues. Of the two targeting elements, the effect of magnetic guidance was shown to dominate arterial localization (*p* = 0.004 vs. 0.084 for magnetic targeting and peptide modification, respectively), consistent with the magnetically driven MNP accumulation step defining the extent of the ultimate affinity-mediated arterial binding and subsequent retention of the carrier particles. The enhanced arterial uptake and sustained presence of paclitaxel-loaded MNPs at the site of stent deployment were associated with a strong inhibition of restenosis in the rat carotid stenting model, with both the neointima-to-media ratio (N/M) and % stenosis markedly reduced in the dual-targeted treatment group (1.62 ± 0.2 and 21 ± 3 vs. 2.17 ± 0.40 and 29 ± 6 in the control animals; *p* < 0.05). We conclude that the dual-targeted delivery of antirestenotic agents formulated in fibrin-avid MNPs can provide a new platform for the safe and effective treatment of in-stent restenosis.

## 1. Introduction

Restenosis is a serious complication of angioplasty procedures that are applied to relieve atherosclerotic obstruction in coronary and peripheral vasculature. Vascular stents, first used only as metallic scaffolds, and later redesigned into a platform for pharmacotherapy (namely, drug-eluting stents, or DES), have substantially reduced the incidence of restenosis in coronary artery disease patients with non-complex lesions [1,2,3]. However, the finite drug payload and inability to adjust the drug dose to complex settings presented by real-world patients are significant limitations of DES, often leading to a particularly challenging complication, “DES restenosis” [2,3,4]. In this study, we developed and characterized a new therapeutic strategy for preventing the reocclusion of injured blood vessels (restenosis) using the magnetically guided delivery of biodegradable nanoparticles endowed with a strong affinity for the injured arterial wall via their surface modification with a fibrin-avid peptide.

Our lab previously demonstrated the feasibility of a novel magnetic guidance approach, taking advantage of vascular stents for magnetically guided, site-specific delivery [5], and this targeting scheme was later successfully used in a pig model of in-stent thrombosis, confirming experimentally its applicability to human-sized blood vessels [6]. Practically, we showed that a 1000 G uniform field is sufficient for the near-maximal magnetization of stents made of 304-grade stainless steel, a paramagnetic alloy that has a history of safe use in implantable cardiovascular devices [7,8]. The application of MNPs concomitant with a brief (5 min) exposure to the uniform field at this strength resulted in the effective and highly site-specific localization of MNPs to the injured artery [1]. Following the magnetic treatment, a four-fold greater MNP amount was detected in stented arteries at the earliest examined time point (5 min), and this ratio between the magnetic vs. non-magnetic treatment groups remained in the 5.5–9.5 range at later time points, up to 5 days post-treatment. While magnetic guidance achieved a high initial localization to stented arteries in comparison to peripheral tissues, similar fractional MNP redistribution rates were observed in magnetically treated and control animals, suggesting that magnetic guidance alone may not provide a mechanism for retaining the carrier at the delivery site—a result expected for superparamagnetic MNPs designed to exhibit no residual magnetization as required for their safe clinical use [9,10]. Based on these findings, the performance of this magnetic targeting strategy, unique in its potential scalability to human patients and applicability to deep blood vessels and effective at localizing MNPs within the stented region, can be further improved by enhancing the interaction of MNPs with the arterial wall, thus extending the drug presence at the target site and minimizing its dissemination. This, in turn, is expected to result in a greater inhibition of restenosis with fewer adverse effects caused by the action of drug-loaded MNPs on healthy organs and tissues.

In the present studies, we evaluated a novel targeting approach integrating the two-source magnetic guidance scheme with fibrin affinity for enhancing and prolonging the association of the drug nanocarriers with the injured arterial wall. These studies were designed to examine the hypothesis that the effect of the site-specific delivery of biodegradable nanocarriers accomplished with magnetic guidance to the stented segment can be enhanced and extended by using a two-stage targeting approach, where the step of the externally controllable physical targeting is followed by the stable anchorage of MNPs in the vicinity of the stent implantation site. The favorable distribution pattern achieved during the brief first stage is thereby retained by taking advantage of the affinity interaction with the fibrin deposited on the luminal surface of the injured arterial wall. Fibrin was selected as a uniquely suited molecular target, both readily accessible to MNPs and spatially restricted to the region of injury and stent deployment.

To examine our hypothesis that the “staged” targeted delivery approach can further improve site-specificity and minimize untoward effects by first actively guiding nanocarriers and then stabilizing their binding to the site of arterial injury, we first demonstrated that MNPs can be endowed with fibrin binding capacity by particle surface modification with a short proline-rich peptide exhibiting a high affinity for the C-terminal portion of the γ-chain of fibrin (GPRPP). This peptide was previously applied in the context of thrombus detection and for the monitoring of thrombogenesis and fibrinolysis [11,12,13,14,15,16] and was shown to bind fibrin with high avidity due to >10^15^ binding sites per mg of fibrin protein [11]. The high avidity and the multivalent binding mechanism of peptide-functionalized MNPs can both be instrumental for extending the retention of the magnetic carriers at the site of stent placement.

Our aims were to optimize this conceptually novel strategy using biodegradable MNPs post-modified with the fibrin-avid peptide as a targeting ligand in combination with two-source magnetic guidance, and to evaluate the utility of this targeting approach in the context of site-specific drug delivery to stented blood vessels. Peptide-modified MNPs applied to rat carotid arteries using a magnetizing uniform field exhibited a sustained presence in the stented arterial segment at levels greatly exceeding those achievable in the absence of affinity modification consistent with prolonged retention of magnetically directed particles at the target site. While paclitaxel was chosen here as a model antirestenotic agent, both the formulation methodology and staged targeting-based delivery can potentially be applied to different classes of small-molecule drugs and biopharmaceuticals, providing the basis for safer and more effective new treatment modalities for treating restenosis and other proliferative vascular diseases.

## 2. Materials and Methods

### 2.1. Nanoparticle Formulation and Surface Functionalization

Poly(D,L-lactide)-based MNPs colloidally stabilized with bovine serum albumin were formulated with the inclusion of nanocrystalline magnetite and paclitaxel (PTX) using a modification of the emulsification–solvent evaporation method, as reported previously by our group [1]. In brief, nanocrystalline magnetite formed by alkaline precipitation was first coated with oleic acid. The obtained dispersion of colloidal magnetite in chloroform was used to co-dissolve poly(D,L-lactide) (Mw 122 kDa, Mn 77 kDa, Lakeshore Biomaterials) and PTX. The organic phase was emulsified by sonication in a 1% *w*/*v* aqueous solution of human serum albumin (Octapharma AB), and the solvent was removed under reduced pressure. For fluorescent imaging and fluorimetric analysis, the MNPs were stably labeled with a polylactide-BODIPY_558/568_ conjugate synthesized by coupling poly(D,L-lactide) equipped with a glycinate spacer and BODIPY_558/568_ succinimidyl ester, as previously reported [17]. To quantify MNP surface-associated albumin, an albumin-BODIPY FL conjugate was admixed with plain bovine serum albumin in the aqueous phase at a 1:50 ratio during the particle formulation step. The labeled albumin derivative was determined fluorimetrically (λ_ex_/λ_em_ = 485 nm/535 nm) following MNP digestion with acetonitrile and albumin extraction into a 5M solution of sodium chloride in water.

Albumin-coated MNPs were further functionalized with a fibrin-avid peptide, GPRPP, in its cysteinated form, extended with a flexible GGG linker (purity: ≥90%, Bachem, Bubendorf, Switzerland), using a two-step process: 1.0 mg of sulfosuccinimidyl-4-(N-maleimido-methyl)cyclohexane-1-carboxylate (sulfo-SMCC) was dissolved in 100 µL of sterile water and added dropwise to 1 ml of MNP suspension. The MNPs were incubated for 60 min at 4 °C, washed twice by magnetic decantation, and resuspended in degassed double-distilled water. One mg of the thiolated affinity peptide was dissolved in 100 µL of degassed water and added dropwise to the MNPs. Following a 60 min incubation at 4 °C, the MNPs were washed twice by magnetic decantation to remove unbound peptide, resuspended in an aqueous trehalose solution (10% *w*/*v*) as a cryoprotectant, lyophilized, stored at −20 °C, and reconstituted in deionized water before use. Drug and magnetite loading were determined spectrophotometrically against suitable calibration curves following MNP digestion with hydrochloric acid or two-step drug extraction in *sec*-butanol, respectively, as previously described [18]. Particle size distribution (presented as intensity) was measured by dynamic light scattering. Hysteresis measurements showing a near-superparamagnetic behavior and strong magnetization of MNPs at 1000 G were performed using an alternating gradient magnetometer (Princeton Instruments Corporation, Trenton, NJ, USA).

### 2.2. Quantification of Reactive Amino and Maleimido Groups

Reactive amino and maleimido functions accessible for MNP surface functionalization were determined fluorimetrically using N-hydroxysuccinimide ester and mercaptan derivatives of fluorescein, respectively. Following the incubation with the amino- or thiol-reactive fluorescent probes, the MNPs were repeatedly washed by magnetic decantation to fully remove unbound fluorescein and were degraded by incubation with aqueous sodium hydroxide (1M) at 37 °C for one hour. Following centrifugation, the fluorescein was analyzed in the supernatant at λ_ex_/λ_em_ = 485 nm/535 nm against calibration curves prepared with the respective derivatives. Fluorescein sodium salt was used as a control lacking thiol reactivity to determine non-specific binding.

### 2.3. MNP Disintegration Studies

MNP disassembly was monitored in fetal bovine serum based on changes in the emission spectra of BODIPY_558/568_-labeled MNPs. A correlation between the emission spectra of fluorescently labeled MNPs and their degradation status was first established in accelerated degradation experiments using proteinase K, previously shown to effectively degrade aliphatic polyesters, including polylactide [19]. A mixture of MNPs diluted 1:100 in PBS and combined with proteinase K (150 µg/mL) was incubated at 37 °C with constant stirring. At predetermined time-points, the emission spectrum of the MNP suspension (λ_ex_ = 540 nm) was measured without prior separation, and the ratio between emission intensities at 612 nm and 575 nm was calculated. The integrity status of MNPs was quantified from the fluorophore distribution into a soluble fraction determined after passing the particle suspension through a filter membrane with a 0.02 µm pore size (Anotop, Whatman, Clifton, NJ, USA) impermeable to MNPs.

For measuring MNP disassembly, particle samples were incubated in triplicates at 37 °C with constant mixing, and their F_612_/F_575_ emission ratio was assayed at predetermined time points. The extent of MNP disassembly at given time points was obtained from the F_612_/F_575_ ratio, using the correlation established above.

### 2.4. MNP–Fibrin Binding Analysis

For fibrin coating, wells of a 96-well plate or 0.5 mm glass beads were first treated sequentially with fibrinogen (10 mg/mL at 4 °C overnight) and thrombin (25 U/mL at 37 °C for 30 min), then washed with fetal bovine serum. In **protocol A**, GPRPP peptide-modified or unmodified MNPs stably labeled with BODIPY_558/568_ were applied to the wells with/without a high-gradient magnetic field (32.5 T/m average field gradient). The MNPs retained after 5 min in fibrin-coated and uncoated (control) wells were quantified fluorimetrically (λ_ex_/λ_em_ = 540/575) after digesting the particles with acetonitrile. In **protocol B**, MNPs were incubated with fibrin-coated or uncoated glass beads for 1 h at 37 °C on a rotator. The beads were examined for MNP binding by fluorescent microscopy, and the amount of unbound MNPs in suspension was analyzed fluorimetrically as above.

### 2.5. In Vitro Cell Growth Inhibitory Effects of MNP on Cultured Arterial Smooth Muscle Cells

The effect of MNPs on the viability and growth of cultured smooth muscle cells was examined as a function of dose, magnetic exposure, and surface modification with the GPRPP peptide, using the A10 cell line derived from the thoracic aorta of an embryonic rat and exhibiting the defining characteristics of neointimal smooth muscle cells [20]. The cells were seeded at 5% confluence on 96-well plates. The effect of MNPs on cell proliferation was examined in the presence or absence of a static, high-gradient magnetic field as follows. Varying amounts of PTX-loaded MNPs diluted in DMEM supplemented with 10% FBS were added to the cells for 5 min with/without magnetic exposure (LifeSepTM 96F magnetic separator, Dexter Magnetic Technologies, Elk Grove Village, IL, USA). The Alamar Blue viability assay was performed seven days post-treatment, using untreated cells as a reference.

### 2.6. In Vivo Fluorescent Imaging, Biodistribution Analysis, and Therapeutic Efficacy Evaluation

Angioplasty stenting surgeries were performed on male Sprague Dawley rats, following a previously reported protocol [1]. The left common carotid artery was injured by three passages of a Fogarty catheter before the deployment of a 304-grade stainless steel stent (8 mm). A catheter was introduced via the external carotid into the common carotid artery and positioned distal to the stent. A uniform field (1000 G) was generated across the stented region using paired electromagnets. MNP suspension was delivered through the catheter to the stented arterial segment for over 1 min without interrupting the blood flow (flow-through settings), and the magnetic field was maintained for an additional 10 min before closing the wound. Alternatively, MNPs were applied into a temporarily isolated stented segment of the common carotid artery (dwell delivery settings). After 60 s, ligatures were released, restoring the blood flow, and the magnetic field was maintained across the stented region for an additional 15 min. Animals in the non-magnetic control group were treated as above without magnetic field exposure.

Live animal fluorescent imaging was performed 3 days post-surgery using an IVIS in vivo imaging system. The MNP-associated fluorescent signal, expressed as radiant efficiency, was determined in the stented arterial segments using λ_ex_/λ_em_ of 535 nm/580 nm and 535 nm/620 nm. An MNP analysis was carried out on tissues harvested 7 days post-surgery (≥5 animals per group, including dual- and single-targeted delivery cohorts and animals treated without targeting), including the stented and contralateral arteries, liver, spleen, lungs, and kidneys. PLA-BODIPY_558/568_ was extracted from homogenized tissue samples in acetonitrile in the presence of sodium chloride (5M). The extracted polymer was determined in the supernatants fluorimetrically against a suitable calibration curve (λ_ex_/λ_em_ = 540/575 nm). For therapeutic efficacy evaluation, the rats were euthanized after 28 days, and morphometric measurements were performed on the stented carotid arteries in a blinded fashion (10 animals per group), using a “no treatment” group as a control.

A non-parametric Kruskal–Wallis one-way ANOVA was used to compare the biodistribution results between the animal groups. A two-factor ANOVA was applied to estimate the relative contributions of magnetic guidance and affinity targeting. The differences were termed significant at *p* < 0.05.

## 3. Results and Discussion

### 3.1. Affinity Peptide-Functionalized Magnetic Nanoparticles: In Vitro Characterization

**Figure 1:**
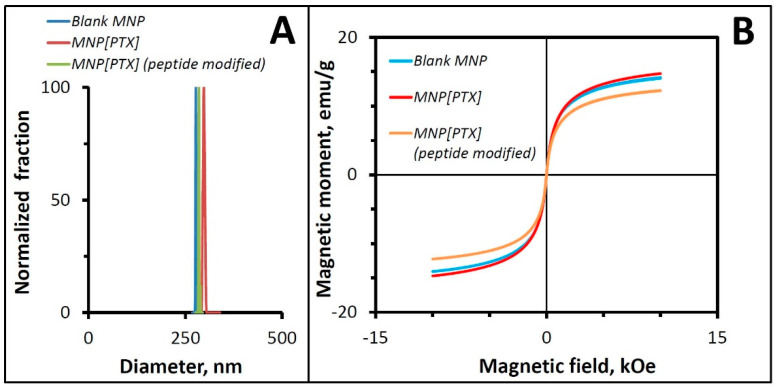
Size distribution and magnetic hysteresis curves of MNPs. MNPs with/without drug or surface functionalization exhibited a narrow size distribution with an average hydrodynamic diameter of 260–290 nm (**A**), strong magnetization at practically achievable field intensities, and a superparamagnetic behavior in the absence of significant remanent magnetization (**B**).

Polylactide-based biodegradable MNPs were formulated with the respective loadings of 40 ± 1% and 4.9 ± 0.5% (*w*/*w*) for magnetite and PTX, and a uniform size distribution (average size: 260–290 nm; PDI: 0.066). In accordance with their high iron oxide loading, the MNPs demonstrated strong magnetic responsiveness. Their previously shown composite structure, where ultrasmall magnetite crystals were embedded in the polymeric matrix [1], was consistent with the absence of significant remanence (remanent magnetization ≤ 1%, Figure 1), enabling strong yet fully reversible magnetization sufficient for effective guidance to arterial stents in vivo.

MNP derivatization with the GPRPP peptide engaging, on average, 12 out of 62 potential binding sites on each surface-associated albumin molecule (accompanied by a shift in the MNP zeta potential from −13.7 ± 0.9 to −6.4 ± 0.9 mV as expected based on the presence of a charged arginine residue in the peptide sequence) was achievable without adversely affecting MNP colloidal stability, size distribution, drug loading, magnetic properties, and capacity for lyophilization.

**Figure 2:**
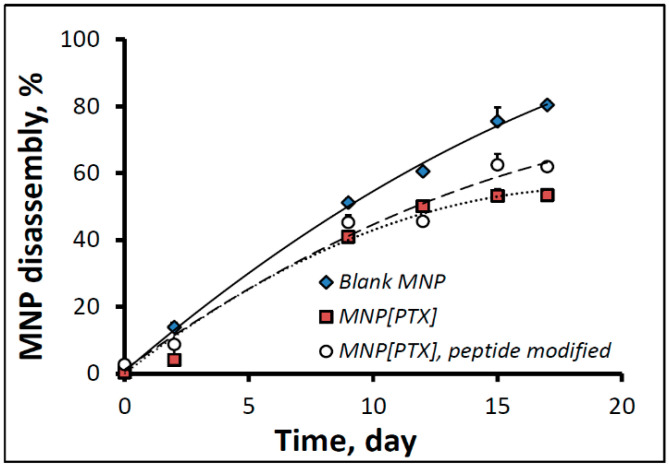
Disassembly kinetics of MNPs in fetal bovine serum. BODIPY_558/568_-labeled blank and PTX-loaded MNPs with or without peptide functionalization were incubated in fetal bovine serum at 37 °C. The emission intensity of MNP suspension at specified time points was measured at 612 nm and 575 nm (λ_ex_ = 540 nm) to determine the particle integrity status. Data are shown as mean ± standard deviation.

The MNP disassembly in fetal bovine serum proceeded at a near-constant rate, with MNP[PTX], surface-modified and unmodified, showing 62.0 ± 0.5% and 53.4 ± 0.3% disintegration, respectively, at day 17. In comparison, blank MNPs formulated without PTX exhibited notably faster disassembly kinetics (80.4 ± 1.3% disintegration by this time point, Figure 2). The slower decomposition of the drug-loaded MNPs was presumably due to the hydrophobizing effect of the lipophilic PTX incorporated in the MNP matrix and posing a barrier to the access of water into the particle core, thereby decelerating the degradation of the particle-forming hydrolyzable polyester, poly(D,L-lactide).

Cell growth inhibition studies performed using cultured rat aortic smooth muscle cells demonstrated a strong antiproliferative effect (a 50% and 75% growth inhibition vs. untreated cells at the MNP doses corresponding to 5 and 20 ng PTX/well, respectively) in the presence of a high-gradient magnetic field. Notably, the surface modification of the drug-loaded MNPs did not alter their capacity for magnetically driven cell growth inhibition, as was evidenced by the near-identical antiproliferative effects of the drug carrier particles with/without surface modification on cultured A10 cells; following a 5 min magnetic exposure, the cell growth was inhibited equally effectively by PTX-loaded peptide-modified or unmodified MNPs in comparison to respective MNPs applied under non-magnetic conditions. Notably, no cell growth inhibitory effect was observed with drug-free (blank) MNPs within the entire examined dose range of 120–1920 ng of MNPs per well.

In our preliminary studies, we compared several previously described peptide sequences targeted to different molecular components found in injured arteries. The formation of fibrin deposits on the luminal arterial surface is triggered by vessel wall trauma during the angioplasty procedure. In our experiments, we found that a fibrin-avid peptide, GPRPP, that binds to the C-terminal portion of the γ-chain of fibrin [11,21] is particularly efficient as a targeting ligand when conjugated to MNPs, in its cysteinated and extended form, using a simple two-step process based on thiol chemistry. Our results showed that the use of albumin as an electrosteric colloidal stabilizer enabled the placement on the MNP surface of about 150 thousand reactive amino groups per particle, of which about one quarter (4 × 10^4^ per particle) could be converted to thiol-reactive groups readily accessible for subsequent ligand attachment. MNP surface functionalization was confirmed to be specific, with no detectable binding due to surface adsorption. Interestingly, we found evidence of two distinct mechanisms driving thiol-mediated attachment. However, at 4 °C, the direct maleimido–thiol coupling occurred markedly faster (near-complete within 1 min of incubation) compared to a second process with notably slower kinetics not requiring the presence of maleimido functions and likely involving the cysteine residues of the surface-localized albumin molecules.

**Figure 3:**
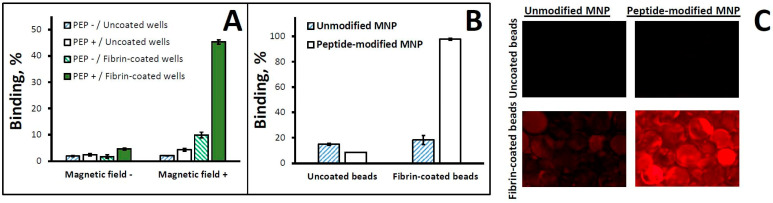
Binding experiments with MNPs modified with fibrin-avid peptide (GPRPP). In (**A**), binding of MNPs to fibrin-coated wells was examined in fetal bovine serum (diluted 1:120 *v*/*v*) as a function of surface functionalization with GPRPP peptide and exposure to a high-gradient magnetic field (average field gradient = 32.5 T/m) using a magnetic separator for 96-well plates. In separate experiments (**B**), depletion of peptide-functionalized MNPs was compared to that of unmodified control MNPs incubated with fibrin-coated glass beads (0.25 mm). Uncoated beads were included as a control. The beads were examined for MNP binding by fluorescent microscopy (original magnification ×40, (**C**)). Results are shown as mean ± standard deviation.

The effectiveness of the fibrin affinity functionalization of MNPs was tested using two complementary binding assays developed in our lab as part of preliminary studies (Figure 3). The affinity interaction of MNPs co-suspended for 1 h with fibrin-coated glass beads resulted in near-complete binding, compared to ≤20% for non-modified MNPs or uncoated beads (Figure 3B,C), confirming the strong fibrin binding capacity endowed by the particle surface modification (*p* < 0.001 by two-factor ANOVA). Furthermore, a brief (1 min) exposure to a high-gradient magnetic field, in combination with particle surface functionalization, drove the rapid and stable immobilization of MNPs on a fibrin-coated substrate (45.3 ± 0.8% binding with combined magnetic/affinity targeting vs. 4.7 ± 0.4% and 10.0 ± 1.1%, respectively, with magnetic exposure or affinity interaction alone, Figure 3A).

### 3.2. In Vivo Targeting, MNP Biodistribution, and Antirestenotic Efficacy

**Figure 4:**
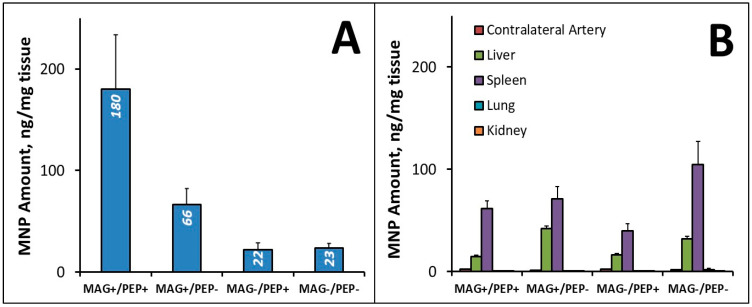
Comparative analysis of MNP levels in stented arteries (**A**) and in non-target tissues (**B**) 7 days after MNP delivery with/without magnetic exposure (MAG+ and MAG−, respectively) or with/without functionalization with the affinity ligand (PEP+ and PEP−, respectively). Note the markedly improved target:non-target ratio in the experimental (MAG+/PEP+) vs. control animal groups. Data are shown as mean ± standard error (n ≥ 5).

We next examined the biodistribution of magnetically guided MNPs surface-modified with the targeting peptide in a rat carotid stenting model established and validated by Finn et al. [22] and more recently adapted by our group for experimental studies [1]. Tissue weight-normalized MNP levels in the stented arterial segments seven days post-treatment are shown in Figure 4A. The dual (magnetic/affinity, MAG+/PEP+) targeting strategy tested using the flow-through settings strongly enhanced MNP localization and retention in the stented arterial segments, with MNP levels significantly (8-fold) higher compared to those in the non-targeted control group (MAG−/PEP−). In contrast, the control groups with one of the two targeting elements omitted (the MNPs either non-functionalized or applied in the absence of the magnetic exposure: MAG+/PEP− and MAG−/PEP+, respectively) were not significantly different from the MAG−/PEP− control. In the absence of magnetic guidance, particle surface functionalization alone did not improve MNP localization to the stented arterial segment (Figure 4A). However, surface functionalization with the fibrin-avid peptide led to a 3-fold difference in the local MNP levels when delivery was carried out in the presence of the magnetizing field. This finding is consistent with the key role of the magnetically driven MNP accumulation, which both precedes and defines the extent of the affinity-mediated arterial binding and subsequent retention of the carrier particles. Consistent with the staged targeting model, the effect of magnetic guidance was shown to be dominant (*p* = 0.004 vs. 0.084 for magnetic targeting and peptide modification, respectively, as per the results of the regression analysis). However, the two targeting elements acted together to maximize the amount of MNPs localized within the stented segment, as suggested by their interaction revealed by the data analysis (*p* = 0.050).

Importantly, the enhanced MNP localization in the stented arterial segment by the dual targeting strategy was paralleled by markedly reduced particle distribution to non-target tissues (Figure 4B). Negligible amounts of MNPs were detected in the contralateral (control) arteries, lungs, or kidneys. Furthermore, the weight-normalized MNP amounts detected in the stented arteries of the dual targeting group’s animals markedly exceeded those found in the spleen and liver (180 ± 53 ng, 62 ± 7 ng, and 14 ± 2 ng per mg of tissue, respectively). Thus, the combined magnetic/affinity targeting substantially improved the target:non-target ratio (14 ± 4 for target/liver and 3 ± 1 for target/spleen) in comparison to the untargeted control (0.8 ± 0.2 for target/liver and 0.3 ± 0.1 for target/spleen), whereas no significant increase in the target:non-target ratios was evident in either of the single targeting groups.

**Figure 5:**
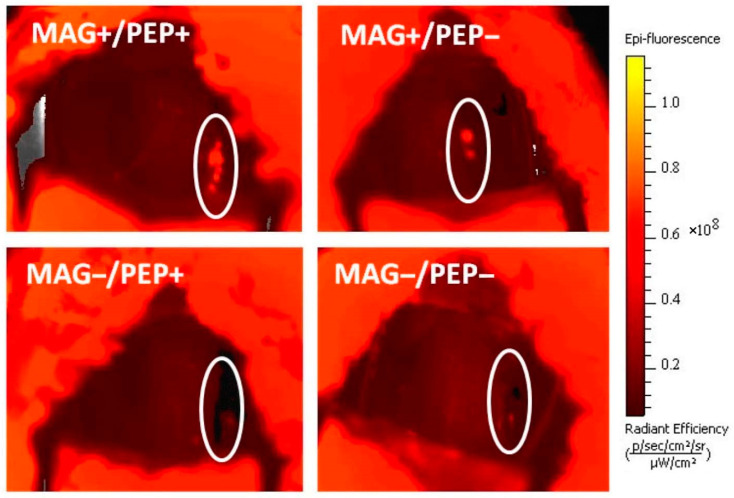
Representative live animal fluorescence images of MNP localization in stented arteries 3 days post-surgery. Stented artery segments are marked on each image.

The results of live animal fluorescent imaging performed 3 days after the procedure (Figure 5) agreed with the fluorimetric analysis findings. Stent-localized MNPs were uniformly observed along the stented segment in the dual targeting group (MAG+/PEP+). A notably lower signal associated with the stented arterial segment was detected in the MAG+/PEP− group, whereas in the two groups treated without magnetic exposure (MAG−/PEP+, MAG−/PEP−), no signal was detectable in the stented artery (See Table 1). Consistent with the results of the biodistribution analysis, magnetic guidance was found to dominate the uptake and retention of MNPs in the stented arterial segment (*p* = 0.003 for the effect of magnetic delivery compared to 0.036 for peptide modification).

Targeting efficiency and selectivity were further enhanced by dwell delivery that additionally reduced the non-target distribution of MNPs while allowing for more extensive contact and association of the particles with the elements of the arterial wall at the target site. It is of note that this dwell delivery approach with a temporary blood flow restriction through the stented artery (over 60 s) is feasible in the clinical setting using standard porous balloons with occlusion catheters. As part of evaluating this delivery protocol modification, we determined the biodistribution of magnetically guided, peptide-functionalized MNPs seven days post-treatment. Both local MNP levels at the site of stent placement and the target:non-target ratios were found to be further improved (78 ± 25, 47 ± 15, and [2.4 ± 0.8] × 10^4^ for the liver, spleen, and lungs) in comparison to the respective values of 14 ± 4, 3 ± 1, and [0.6 ± 0.2] × 10^4^ determined at this time point in the animals treated using the unrestricted flow-through conditions.

In agreement with the enhanced target specificity and prolonged retention, a marked reduction in arterial restenosis was observed in the experimental animals treated with PTX-loaded MNPs using dual-targeted delivery: both the neointima/media (N/M) ratio and % stenosis were found to be strongly reduced in this group, with respective values of 1.62 ± 0.2 and 21 ± 3 vs. 2.17 ± 0.40 and 29 ± 6 in the control animals (*p* = 0.03 and 0.04 for N/M and % stenosis). The antirestenotic efficacy of the combined magnetically guided/affinity-targeted treatment was also greater than that seen with either targeting component alone or with MNPs delivered without targeting. Collectively, these results suggest that surface functionalization endowing particles with fibrin affinity can be used cooperatively with magnetic guidance to prolong the arterial tissue exposure to MNP-encapsulated agents. In addition to increasing and extending the local presence of the carrier particles, which is key to achieving a stronger response at a low total drug dose, the dual targeting strategy also substantially reduces dissemination to non-target tissues, thereby minimizing potential untoward effects.

### 3.3. Nanocarrier-Based Delivery for Site-Specific Restenosis Therapy

While the delivery of antiproliferative agents from vascular stents has reduced the incidence of restenosis in coronary artery disease patients with non-complex lesions [23], the long-term safety of DES remains compromised by delayed healing, hypersensitivity, and local inflammation, all contributing to neointimal hyperplasia and neoatherosclerosis [24]. As a result, the long-term risk of in-stent restenosis remains significant even with the newest DES devices available today [2]. Due to a finite drug payload that cannot be replenished once exhausted and the inability to adjust the drug delivery profile to the anatomy, size, and disease status of a treated blood vessel, the therapeutic performance of DES in complex settings is often found to be suboptimal [25]. Therefore, despite the ongoing development and optimization of stent technology, a need remains for conceptually different drug delivery strategies offering greater therapeutic efficacy and safety.

Stent-targeted delivery of therapeutic agents formulated in biodegradable nanocarriers has the potential to address these challenges [9]. Here we evaluated the feasibility of a site-specific delivery strategy combining magnetic guidance with affinity binding to enhance the localization and retention of nanocarriers in stented arteries. The two-source magnetic targeting scheme we used to guide magnetizable particles to the region of stent implantation is distinct from the commonly used single-source approaches, as it is applicable to non-superficial sites via a coordinated action of two field sources: a primary source, such as an MRI scanner or a magnetic navigation system, which provides a strong, uniform, and deep-penetrating magnetizing field, and a secondary (dependent) source, such as a vascular stent made of a reversibly magnetizable alloy, positioned in the injured arterial segment and focusing the magnetic force by creating a region of highly localized and strong field gradients [26]. It is noteworthy that, in this experimental approach, neither the stent nor MNPs need to be permanently magnetic or have any significant magnetic memory. As the stent configured in thin intersecting struts becomes magnetized, its geometry makes it highly efficient for guiding MNPs to the region of stent deployment by creating strong field gradients in its vicinity [1].

Magnetic guidance used for initial MNP localization can be complemented with affinity binding to achieve stable retention through enhancing the interaction of the particles with the arterial wall, thus extending their presence at the target site. The feasibility and effectiveness of this combined physical/affinity targeting approach are supported by the results of the present studies. To endow MNPs with a binding affinity for injured blood vessels, we employed thiol chemistry-based particle surface functionalization with a fibrin α-chain N-terminal short peptide that binds to the C-terminal portion of the fibrin γ-chain [21]. This peptide was previously investigated and shown to be effective for the magnetic resonance-based detection and monitoring of thrombogenesis and fibrinolysis [11,13]. However, its application for stabilizing anchorage of therapeutic nanocarriers at sites of arterial injury has remained unexplored. Together with the high modification yield of MNPs capable of accommodating 4 × 10^4^ peptide moieties per particle, the high avidity and the multivalent binding mechanism of this peptide enable the stable binding of the nanocarriers to the site of stent placement, resulting in a several-fold increase in MNP levels in stented arteries 3 and 7 days post-treatment. The marked enhancement in site specificity and the potent mitigation of the injury-triggered arterial reocclusion observed in our study warrant further development and optimization of this dual-targeted delivery strategy using surface-functionalized magnetic nanocarriers as a promising new modality for treating in-stent restenosis.

## 4. Conclusions

We demonstrated the feasibility and effectiveness of magnetic guidance combined with affinity-driven arterial binding and the retention of drug-loaded nanocarriers in stented arteries as an experimental targeted delivery approach for in-stent restenosis therapy. The dual magnetic/affinity targeting strategy was shown to both effectively enhance the localization and anchorage of MNPs in stented arteries and to markedly lower the dissemination of the drug carrier to peripheral organs in a rat carotid stenting model. Integrating magnetic guidance capacity with affinity ligand functionalization in the design of biodegradable nanocarriers, shown here to improve the arterial delivery of a small-molecule antirestenotic agent, can potentially be extended to a variety of molecular cargoes, including therapeutic enzymes and gene vectors, paving the way to safer and more effective new therapies for vascular disease.

## Figures and Tables

**Table 1:** Quantitative analysis examining presence of BODIPY_558/568_-labeled MNPs in stented arteries 3 days after treatment with/without applying the affinity ligand modification or exposure to the magnetic field. Data are shown as mean ± standard error (n ≥ 5).

Targeting Strategy	Radiant Efficiency, ×10^6^
MAG+/PEP+	47.9 ± 13.7
MAG+/PEP−	9.6 ± 7.0
MAG−/PEP+	0.6 ± 4.4
MAG−/PEP−	−0.4 ± 2.8

## Data Availability

Data are contained within the article.
